# Lengthening the Lower Extremities of Children with Ollier’s and Maffucci’s Enchondromatosis Using Implantable Lengthening Nails

**DOI:** 10.3390/children8060502

**Published:** 2021-06-14

**Authors:** Aaron J. Huser, Jason Shih Hoellwarth, Valentino Coppa, David S. Feldman, Dror Paley

**Affiliations:** Paley Institute, West Palm Beach, FL 33407, USA; jason@paleyinstitute.org (J.S.H.); coppa.valentino@gmail.com (V.C.); dfeldman@paleyinstitute.org (D.S.F.); dpaley@paleyinstitute.org (D.P.)

**Keywords:** implantable nail lengthening, motorized nail lengthening, lengthening nail, enchondromatosis, Ollier, Maffucci

## Abstract

There are multiple forms of enchondromatosis with Ollier’s and Maffucci’s being the most prevalent types. Limb length discrepancy is a common problem in patients with Ollier’s and Maffucci’s enchondromatosis. There are multiple reports about lengthening bones in patients with enchondromatosis using external fixators. However, there are no case series regarding the use of implantable lengthening technology. The purpose of this paper is to describe our experience with implantable nail lengthening in patients with enchondromatosis. A retrospective chart and radiographic review of patients with enchondromatosis who underwent implantable nail limb lengthening was performed. Seven patients with 14 bony segments were reviewed. A total of 11/14 lengthenings were completed without difficulty. There were no issues in terms of fixation location in patients with Ollier’s disease. One patient with Maffucci’s syndrome experienced migration of the nail during two lengthenings due to a combination of intralesional fixation and preconsolidation. One patient with Ollier’s disease developed a knee extension contracture requiring manipulation under anesthesia. No other complications were recorded. The use of implantable nail lengthening to resolve limb length discrepancies in patients with Ollier’s disease appears to be safe and effective.

## 1. Introduction

Enchondromas are benign, cartilaginous tumors that form in the intramedullary canal of bones and are formed by physeal chondrocytes that did not transdifferentiate into osteoblasts [1]. Enchondromatosis is defined as multiple enchondromas in the bones. Ollier’s disease, first described over 120 years ago, is a form of enchondromatosis with asymmetric distribution and multiple, usually benign, cartilaginous tumors in the metaphyseal regions of the long bones [2]. These enchondromas are also commonly found in the hands and feet. Spranger et al. further classified enchondromatosis into six different types based on radiographic findings and anatomic distribution [3]. Ollier’s disease, the most common enchondromatosis with a prevalence of 1/100,000 [4], is Spranger type I. Maffucci’s syndrome (type II), which features associated hemangiomas, and metachondromatosis (type III) characterized by a combination of exostoses and enchondromas, are less prevalent than Ollier’s disease. Type IV (spondyloenchondrodysplasia), type V (dyssplondyloenchondromatosis), and type VI (cheirospondyloenchondromatosis) are even more rare and involve enchondromas in the spine [5]. Although the inheritance patterns for Ollier’s disease are unclear, recent studies have found mutations in the parathyroid-related peptide type 1 receptor (PTHR1) and the isocitrate dehydrogenase gene (IDH) [5,6]. Zhang and Alman reviewed genetic findings in enchondromatosis and found somatic mutations in IDH1/2 to be the most common abnormality and suggest that pharmacologic therapies could target this gene or downstream pathways affected by the mutation [1].

Patients with Ollier’s and Maffucci’s enchondromatosis often develop coronal and sagittal plane skeletal malalignment and limb length discrepancies (LLD) in both the upper and lower extremities [7]. Fractures through the tumors may also induce deformity and LLD [4]. Indications for treatment of lower LLD include symptomatic LLD or lower LLD > 2 cm [8]. Treatment options include shoe lift, epiphysiodesis (if the patient has adequate growth remaining), bone shortening, limb lengthening, or amputation. Historically, external fixation has been the modality of choice for limb lengthening up until the introduction of implantable lengthening [9]. Previous studies have reviewed lower extremity limb lengthening with external fixation, but only case reports discuss using implantable lengthening nails [10,11,12].

The purpose of this series was to present our experience with lower limb lengthening using implantable lengthening nails for patients with Ollier’s and Maffucci’s enchondromatosis.

## 2. Materials and Methods

Upon institutional review board approval, a retrospective chart and radiographic review was performed on all patients diagnosed with Ollier’s disease, Maffucci’s syndrome and/or enchondromatosis since 2012. Patients were included in this study if the diagnosis of enchondromatosis was confirmed via radiographic review and had undergone lower limb lengthening with implantable lengthening nails (Precice or Precice Stryde, Nuvasive Inc., San Diego, CA, USA). Patients were excluded if radiographs were not available for analysis or if the lengthening was performed using an external fixator or an implantable lengthening plate.

The surgical techniques have been previously described for both the femur and the tibia [13,14]. The bone lengthened was chosen based on the discrepancy compared to the contralateral side. All nails were inserted antegrade. Patients typically stay in the hospital 1–2 nights and then are discharged home. Lengthening is started 3–7 days following the nail insertion. Patients follow-up with their surgeon every 1–2 weeks for a radiographic and clinical check to ensure they are not developing issues such as muscle contracture or joint instability, the nail and bone are lengthening, the newly formed bone (regenerate) is healthy appearing. The postoperative rehabilitation protocol includes partial weight bearing based on the size of the nail (usually 50–70 pounds) and a standardized outpatient therapy regimen 5–6 times/week. The focus of physical therapy is maintenance of joint motion above and below the bone that was operated on. The therapist also instructs the patient on strengthening exercises and develops a program that the patient can do at home to supplement the outpatient therapy. Upon achieving the desired length, patients return home and have monthly radiographic evaluation to ensure the regenerate (newly forming bone at the lengthening site) consolidates and no alignment or hardware issues occur.

Data obtained from the chart included age at the time of nail insertion, sex, lengthening rate and complications. Radiographs were assessed to determine location of distal and proximal locking hardware immediately postoperative and again at the final follow up, corticotomy location, individual bone length, LLD and length achieved. Corticotomy and cross-locking screw position were classified as extralesional (completely outside pathologic bone), intralesional (in an enchondroma), or transitional, as defined by Goote et al. [15]. Healing index was defined as months per cm [16]. Descriptive statistics were calculated.

Each patient had one of two goals for their lengthening. If their LLD was greater than the nail’s maximum length capacity (50 or 80 mm), the goal was to achieve the nail’s maximum lengthening ability. If their LLD was less than the nail’s maximum, the goal was to equalize the limbs. Because there is no way to determine the amount the nail has actually lengthened in situ, assessment of lengthening is performed by noting the external remote control (ERC)-instructed lengthening and by radiographic evaluation. There are unavoidable inaccuracies to both methods [17]. The ERC sends magnetic impulses to impart motion to the nail’s gears; these impulses may not always perform the exact lengthening instructed due to interference from soft tissues, improper ERC placement, or resistance of soft tissues to the lengthening force. Radiographs are subject to magnification inaccuracies, and despite meticulous patient and magnification marker placement, assessing sizes remains inexact. Therefore, we consider nails to have achieved their maximum length when the radiographs measure within 5 mm of the goal length, and the ERC meets or exceeds the implant’s maximum (there has been no implant damage reported to occur when attempting to lengthen 2–3 mm beyond the nail’s maximum; the implant simply does not lengthen anymore).

## 3. Results

Twenty-five patients were identified with the diagnosis of Ollier’s disease, Maffucci’s syndrome or enchondromatosis. Seven patients underwent fourteen lower limb lengthening procedures with implantable lengthening nails. The cases are summarized in Table 1. Six patients were diagnosed with Ollier’s disease and a seventh patient, after undergoing the first lengthening, developed hemangiomas and was diagnosed with Maffucci’s syndrome. There were two females and five males. Four of the patients had right lower limbs that were short and three of the patients had left lower limbs that were short. The mean age was 10.8 years (±3.4 years) at the time of surgery. Twelve of the limb segments were femurs and the remaining segments were tibias. The two tibial segments were in the same patient and lengthened at different times. The mean preoperative leg length discrepancy was 92 mm (±28 mm). The mean preoperative femoral discrepancy was 66 mm (±22 mm) and the mean preoperative tibial discrepancy (of all limbs) was 32 mm (±23 mm). The mean lengthening rate for the femurs was 0.9 mm/day (±0.14 mm/day). Both tibias were lengthened at 1 mm/day. The mean femoral length achieved was 54 mm (±20 mm). Both tibial lengthenings achieved 50 mm. Nine femoral segments had data available for the healing index. The mean healing index for the femoral segments was 0.9 mo/cm (±0.3 mo/cm). The healing index for one tibia was 1.00 mo/cm and the other was 0.92 mo/cm.

**Figure 1:**
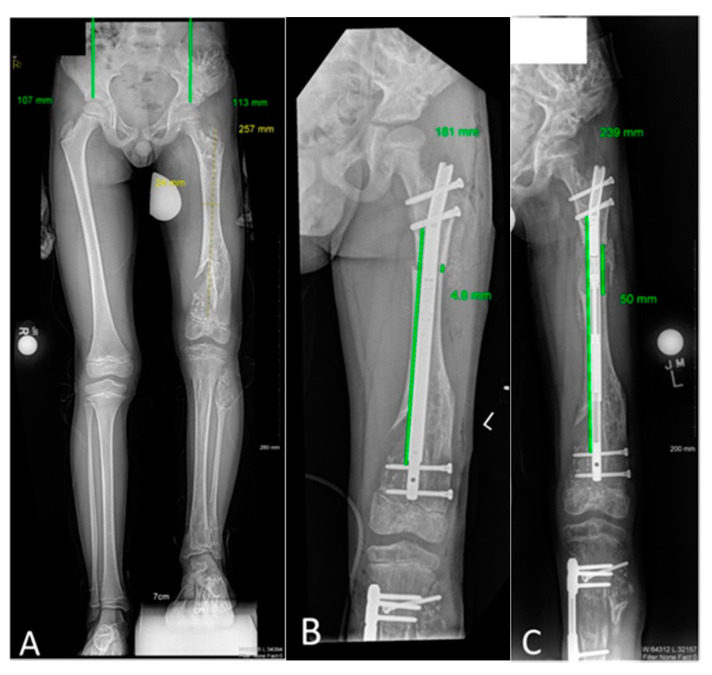
Uncomplicated limb lengthening episode with a motorized intramedullary nail for a patient with enchondromatosis. This figure portrays Patient 6’s lengthening experience. A 9-year-old male with Ollier’s disease affecting his left femur (proximal and distal metaphyseal), tibia (proximal metaphyseal), and fibula (head-neck region). (**A**) The patient had an initial right > left leg length difference of 76 mm. (**B**) He was managed with left femur osteotomy and implantable lengthening nail. The proximal and distal cross-locking screws were placed intralesionally. He also had ipsilateral tibia osteotomy and lengthening with a implantable lengthening plate, which is not the focus of the current manuscript. (**C**) After 75 days, the femur nail had achieved its maximum excursion (50 mm) with corresponding increase in the femur osteotomy gap. Appropriate early regenerate can be observed. This patient had an uncomplicated lengthening experience and is currently consolidating the femur, tibia, and fibula sites.

**Figure 2:**
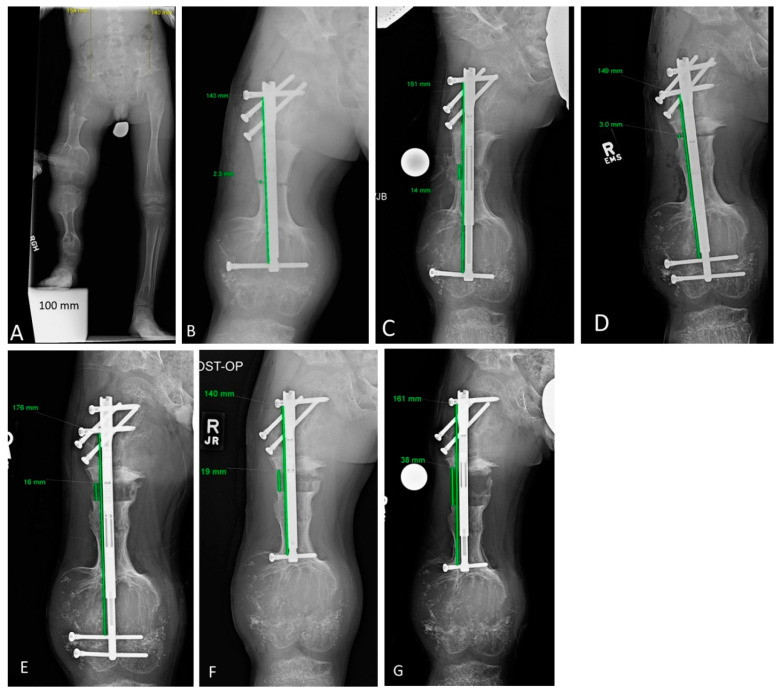
Limb lengthening episode complicated by mismatched hardware and osteotomy distraction. This figure portrays Patient 5’s lengthening experience. A 7-year 3-month-old male with enchondromatosis affecting all long bones of both legs, most severely the right femur and tibia. The initial diagnosis was Ollier’s disease. (**A**) His first lengthening was performed to address left greater than right leg discrepancy of 114 mm. (**B**) He was managed with right femur osteotomy and implantable lengthening nail (Precice). The angled femoral neck screws are anterior to the nail and were placed to prophylactically reinforce the femoral neck. The proximal and distal cross-locking screws are intralesional. (**C**) The nail eventually elongated approximately 40 mm, while the osteotomy gap elongated only approximately 12 mm, which along with the noticeable difference of the distal cross-lock screw position relative to the condyles, indicated the distal intralesional fixation provided insufficient purchase. The patient had never experienced any pain or other complications other than the mismatch between nail elongation and osteotomy distraction. (**D**) Given there was no need for emergent intervention, the patient was given a consolidation period of one year and lengthening was attempted again. A longer nail was inserted and cross-locking screws were placed on either side of the distal physis. (**E**) Premature consolidation and nail migration was redemonstrated; there was a nearly 2:1 nail:osteotomy elongation mismatch. (**F**) Fixation was changed to a shorter nail which, although allowing only one distal cross-locking screw, it was placed in extralesional bone and intra-operatively felt stronger during drilling and insertion. (**G**) The nail and osteotomy gap then lengthened proportionately; he eventually achieved 47 mm of distraction and was stopped due to proximal screw migration. While this patient remained under postoperative lengthening observation, his diagnosis evolved from Ollier’s disease to Maffucci’s syndrome, based on the development of hemangiomas.

Eight of our osteotomies were extralesional and six were transitional. All but one was healed at the time of publication with radiographically normal bone. The one that was not healed had just completed lengthening without complication. Twenty-five of the proximal locking screws and twenty-one of the distal locking screws were placed intralesionally. Two proximal and three distal locking screws were placed extralesionally. One proximal and four distal locking screws were placed in a transitional zone. All but one patient lengthened without fixation issues (Figure 1). The only patient, with the diagnosis of Maffucci’s syndrome, that experienced hardware migration had both the proximal and distal interlocking screws were intralesional (Figure 2). The initial lengthening was halted and a year later, the patient was taken back to the OR. Two proximal screws were placed intralesionally, one distal locking screw was placed in a transitional location and one distal screw was placed in an intralesional location. The patient experienced a second migration and the nail was replaced with a shorter nail, this time with an intralesional proximal screw and extralesional distal screw. The patient was able to achieve a total of 47 mm of lengthening before experiencing a third migration of the proximal locking screw and lengthening was ceased which was considered the goal. All remaining patients were able to achieve their length without any other hardware-related complications or malfunctions. The only other complication in our series was a knee extension contracture in patient 1 who was undergoing simultaneous femoral lengthening with a nail and tibial lengthening with an external fixator. The lengthening or hardware irritation from the distal locking screws may have caused the extension contracture. The patient was initially treated with slowing/stopping of the lengthening and physical therapy but the contracture did not improve. The patient required manipulation under anesthesia, botulinum injection into the quadriceps muscle, and physical therapy; eventually the patient was able to regain full motion.

## 4. Discussion

This is the first published case series of implantable nail lengthening in patients with Ollier’s disease/enchondromatosis. The most notable observation from our case series is that implantable nail lengthening appears suitable and well tolerated by patients with enchondromatosis, specifically Ollier’s disease and potentially Maffucci’s syndrome.

The available literature regarding outcomes of limb lengthening in patients with Ollier’s disease is sparse. Angelini recently performed a systematic review, analyzing 19 studies, all retrospective, that evaluated limb lengthening in this subset of patients [10]. Ten case reports (≤3 patients), seven case series (≥4 patients), and two cohort studies presented 121 patients (272 bone segments, 13 upper extremity and 259 lower extremity). All but three lower extremity segments were lengthened with an external fixator. Those three segments (one femur twice and one tibia) were all in one patient and were lengthened with an implantable lengthening nail [11].

Overall, the review demonstrated that length was able to be achieved in a timely manner and with similar complication rates (27.9%) to studies of other lengthening patients. Complications included joint stiffness (the most common), infection (mostly related to the pin sites), early healing of the lengthening site, fracture, recurrence of deformity, delayed/non-union, neuropraxia and overlengthening. These complications have been documented in other reviews of lengthening [18,19]. In our series, one patient experienced a knee extension contracture and another patient experienced premature consolidation which led to nail migration. Our series did not have any infections; however, our series is underpowered to make any conclusions about infection. We also did not have any fractures postoperatively which may be due in part to the added strength provided by the retained nail.

An attentive physical examination, radiographic evaluation, and discussion of patient goals are critical to determining the appropriate surgical plan and implants. Traditionally, Ilizarov-style external fixators were used but we prefer to use implantable lengthening nails in the femur and tibia if the bone size and morphology permits. External fixators allow gradual correction in all planes but the transcutaneous fixation can lead to infection and the bulk of the fixator presents a major hassle for patients. A benefit of eliminating the transcutaneous fixation via lengthening nails is to reduce infection risk, specifically pin and wire site infection [20]. Another benefit is improved patient experience without the physically cumbersome and cosmetically unappealing frame. Further, frames are eventually removed after consolidation, leading to a period when patients may fracture due to disuse osteopenia or incompletely mineralized regenerate. While this can be mitigated by inserting flexible nails [21], internal lengthening nails can simply remain in situ and can prevent fracture or serve as already-existing stabilization should a fracture occur.

**Figure 3:**
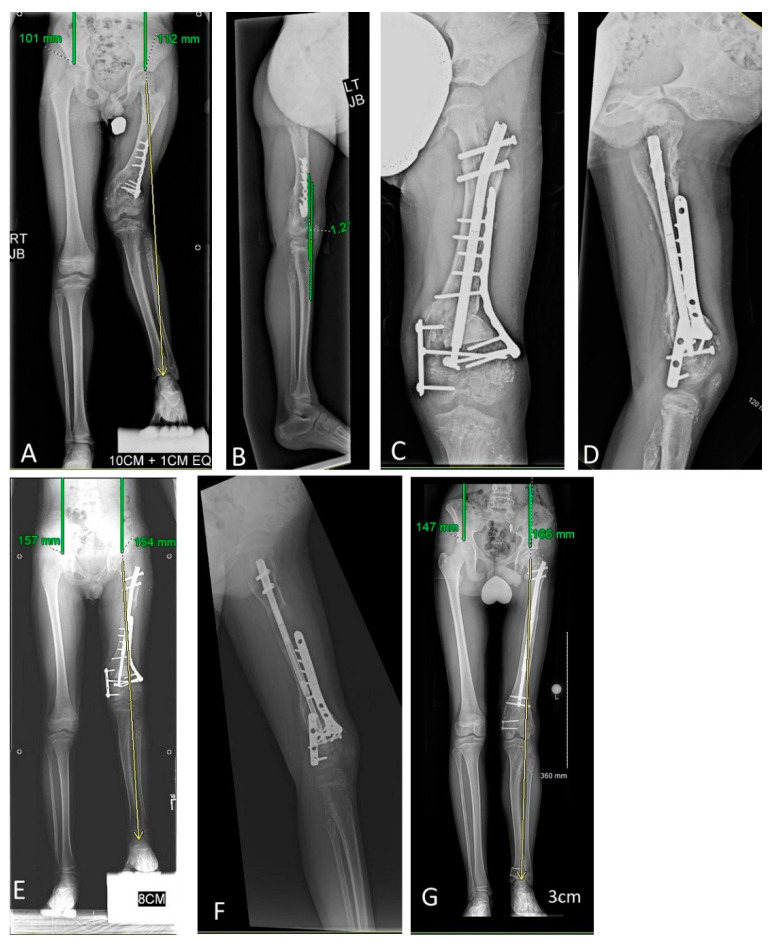
Acute coronal plane deformity correction with simultaneous intramedullary motorized nail lengthening. This figure portrays Patient 7’s first lengthening experience. (**A**) An 8-year old male with enchondromatosis predominantly affecting the left femur, radiographically consistent with Ollier’s disease. Left leg shorter than right by 122 mm, predicted to be 184 mm at maturity. Coronal full leg length radiography identifies additional substantial left genu valgus deformity mainly occurring in the distal femur. (**B**) Lateral radiograph identifies no sagittal plane deformity requiring correction. (**C**,**D**) Anterior–posterior and lateral immediate postoperative radiographs demonstrating acute coronal correction via an osteotomy and plate internal fixation strategy, with simultaneous lengthening nail inserted with a proximal osteotomy where lengthening will occur. (**E**) Full length standing radiograph taken upon completion of 50 mm uncomplicated lengthening. The coronal deformity is improved. (**F**) The patient is suboptimally positioned for the lateral radiograph which was not repeated at the time, but is shown to demonstrate no sagittal plane deformity occurred through the femur during the lengthening process. (**G**) The patient’s current full length radiograph after his third lengthening identifies 41 mm residual LLD, with slight genu valgum progression compared to the initially provided correction. A final lengthening surgery with repeat acute coronal plane correction is planned following skeletal maturity.

A limitation of motorized nails is their design limitation of 5 or 8 cm elongation. However, additional length can be achieved with a short outpatient procedure of additional osteotomy and reloading the nail [13,14,22,23]. Additionally, gradual correction can only occur for length, not rotation or alignment. Acute rotation and deformity correction can be performed during surgery. Synchronous alignment improvement can be achieved acutely with a nail or with a separate implant (Figure 3). If the lengthening nail is being used for acute correction, blocking screws are often necessary to ensure angular deformity does not occur as lengthening proceeds, especially in patients with Ollier’s disease as the enchondromas may allow for more toggling of the nail within the bone. Another option is a metachronous approach by performing the lengthening and deformity correction at different stages. Either is appropriate and based on surgeon comfort and shared decision making with the patient. Deformity correction may also be accomplished by temporary hemiepiphysiodesis. Due to the effect of the enchondroma on the physis, temporary hemiepiphysiodesis may end up acting as an epiphysiodesis. It is recommended to follow these patients closely to ensure the guided growth implant is not worsening the LLD. Lastly, if the intramedullary canal of the femur or tibia is too narrow to fit a lengthening nail, we consider still using the nail and placing it outside the bone but under the skin [24]. An additional static intramedullary nail and blocking screws can prevent unintended malalignment.

For all lengthening patients, postoperative follow up and physical therapy are essential to maximizing length gained and preventing complications. We recommend hands-on physical therapist interaction 3–6 days weekly while lengthening. The goals of therapy are to maintain motion at the joints above and below the lengthening bone, and also to prevent joint contracture, subluxation, and dislocation. Additionally, frequent interaction with an attentive physical therapist can identify problems less obvious to a patient such as neurologic motor or sensory changes and earlier surgeon follow-up can be initiated. Several daily at-home self-guided therapy sessions are also prescribed with the same goals of maintaining mobility. Surgeon physical examination and radiographic assessment are typically scheduled every one to two weeks. The patient is assessed for pain, changes in joint motion or stability, and neurologic status. After approximately 7–10 days following surgery, pain typically is minimal and increased pain should raise concerns of joint instability or that the daily lengthening rate is exceeding the patient’s muscle or nerve limitations. Our standard lengthening rate and rhythm is 0.75–1 mm/day split into at least 3–4 sessions daily. Patient 1 in our series developed an extension contracture during the initial lengthening. Lengthening was slowed and then stopped; however, this patient required a manipulation under anesthesia to help treat the extension contracture.

Patients with Ollier’s disease are at risk for premature consolidation and may require an even faster rate [10], and shorter intervals between serial radiographic evaluations. Patient 5 in our series experienced nail migration and this was likely due to preconsolidation of the regenerate and the force to distract the regenerate exceeded the force the enchondroma was exerting on the cross-locking screws. Conversely, if a patient demonstrates inadequate regenerate or develops stigmata of joint subluxation (reduced motion and pain), the rate is slowed. In our series, no patients with Ollier’s disease experienced premature consolidation and this phenomenon was only seen in the patient with Maffucci’s syndrome.

Unique to patients with enchondromatosis is the effect of the enchondromas on fixation and osteogenesis. Goote et al., who lengthened 40 different bone segments with external fixation, found no difference in healing and radiographic appearance of the regenerate when comparing corticotomies through three regions: intralesional, transitional, or extralesional [15]. We did not have any intralesional osteotomies for lengthening our series; however, all our transitional (6) and extralesional (8) osteotomies healed with radiographically normal appearing bone. Goote et al. also published external fixation indices for lengthening over a nail with an external fixator index of 0.8 mo/cm which is similar to our healing index in femurs [15]. Another author biopsied one patient 15 months following lengthening through an enchondroma and the result was normal bone [25]. No biopsies were performed in our series.

Regarding fixation, Chew et al. performed osteotomies of the distal femur for varus correction and did not report any hardware failure with acute deformity correction and internal fixation [26]. Watanbe et al. also did not report hardware failure with intralesional or transitional external fixation [12]. We had 28 proximal locking screws and 28 distal locking screws used in our series. A total of 90% of the proximal locking screws and 75% of the distal locking screws were intralesional. No patients in our series with Ollier’s disease and intralesional fixation had a problem with fixation. In contrast, our single patient with Maffucci’s syndrome (Patient 5) did have fixation issues, specifically as the nail lengthened, the cross-locking screws migrated within the enchondromas without elongation of the osteotomy site. The bone initially lengthened with intralesional fixation, but as the regenerate preconsolidated, the force placed on the screws and subsequently the enchondromas was overwhelming and the fixation/nail migrated. When the cross-lock location was changed to extralesional bone, lengthening was successful. An alternative to using a shorter nail could be to link the lengthening nail to a plate with the cross-lock screw and then fix the plate to sufficient quality bone [27].

This study is not without limitations. First, this is a retrospective case series and our data are only limited to what is reported in the medical records and available radiograph-ically. Second, although enchondromatosis is a rare disease, this is a small series and an increase in numbers would improve the power of the findings. Third, we do not have any patient or parent-reported outcomes to determine whether the clinical success is matched by patient satisfaction and functionality. A prospective, multi-center approach would be the next step in assessing the implantable, lengthening nails in patients with enchondromatosis.

## 5. Conclusions

Implantable lengthening nails can be used to treat LLD in patients with Ollier’s and Maffucci’s enchondromatosis. In Ollier’s disease patients, intralesional fixation appears to be adequate to allow for distraction, but should be watched carefully for signs of premature consolidation or hardware migration. Concomitant physical therapy plays an important role in the success of this procedure. Implantable devices in this population may obviate the need for external fixators to achieve length.

## Figures and Tables

**Table 1:** Patient summaries. M denotes male and F denotes female.

Patient/Sex	Age (Years)	Bone Lengthened	Limb Length Discrepancy (mm)	Rate (mm/Day)	Length Achieved (mm)	Healing Index (mo/cm)	Goal Achieved (yes or no)	Complication	Management
1/M	15	Femur	127	0.60	34	1.60	no	Knee extension contracture	Manipulation under anesthesia, injection of botox into the quadriceps and physical therapy
	18	Femur	46	1.0	45	0.71	yes	none	n/a
2/M	13	Femur	74	1.0	80	0.65	yes	none	n/a
3/M	9	Femur	76	0.75	50	n/a	yes	none	n/a
4/F	6	Femur	100	0.9	50	1.00	yes	none	n/a
		Tibia		1.0	50	1.00	yes	none	n/a
	10	Femur	125	0.75	80	n/a	yes	none	n/a
		Tibia		1.0	50	0.92	yes	none	n/a
5/M	7	Femur	118	1.0	12	n/a	no	Premature consolidation and nail migration	Lengthening was ceased and femur allowed to heal
	9	Femur	100	1.0	47	0.80	yes	Premature consolidation and nail migration ×2	1. Changed distal fixation to the epiphysis, problem recurred. 2. Changed distal fixation to extralesional bone with substantial lengthening achieved before recurrence of proximal screw migration
6/F	9	Femur	51	1.0	55	0.55	yes	none	n/a
7/M	8	Femur	121	0.75	50	0.92	yes	none	n/a
	10	Femur	95	1.0	80	0.86	yes	none	n/a
	12	Femur	71	1.0	68	0.91	yes	none	n/a

## Data Availability

The data for this study have been provided in the tables in the body of this paper.
